# Preparation and Characterization of Dexamethasone Microemulsion Based on Pseudoternary Phase Diagram

**DOI:** 10.17795/jjnpp-9373

**Published:** 2013-07-20

**Authors:** Eskandar Moghimipour, Anayatollah Salimi, Masoud Karami, Sara Isazadeh

**Affiliations:** 1 Nanotechnology Research Center, Jundishapur University of Medical Sciences, Ahvaz, IR Iran; 2 Department of Pharmaceutics, Faculty of Pharmacy, Jundishapur University of Medical Sciences, Ahvaz, IR Iran

**Keywords:** Dexamethasone, Emulsion, Calorimetry, Differential Scanning

## Abstract

**Background:**

The increased incidence of inflammatory diseases has necessitated the need to search for new topical dosage form of dexamethasone.

**Objectives:**

The purpose of the present study was the preparation and evaluation of novel microemulsion as a topical delivery system for dexamethasone by mixing appropriate amount of surfactant including Tween 80 and Labrasol, cosurfactant such as capryol 90 and oil phase including labrafac lipophile wl-transcutol P (10:1 ratio).

**Materials and Methods:**

The prepared microemulsions were evaluated regarding their particle size, zeta potential, X-Ray scattering, conductivity, stability, viscosity, differential scanning calorimetry (DSC), scanning electron microscopy (SEM), refractory index (RI), pH, and x-ray diffraction (XRD).

**Results:**

The results showed that the maximum oil was incorporated in microemulsion system that contained surfactant to cosurfactant ratio of 4:1. The mean droplet size range of microemulsion formulation was in the range of 5.09 to 159 nm, and its refractory index (RI) and pH were 1.44 and 7, respectively. Viscosity range was 57-226 cps. Drug release profile showed that 48.18% of the drug released in the 24 hours of experiment. Also, Hexagonal, cubic and lamellar structures were seen in the SEM photograph and XRD peak of microemulsions.

**Conclusions:**

This study demonstrated that physicochemical properties and in vitro release were dependent upon the contents of S/C, water, and oil percentage in formulations. SAXS technique and SEM obtained important information about microstructure of microemulsions. W/O and bicontinuous microemulsion with different microstructures were found in formulations.

## 1. Background

The word microemulsion was originally proposed by Hoar and Schulman in the earlier of the 1940s. They generated a clear single-phase solution by titrating a milky emulsion with hexanol (1). Microemulsions are thermodynamically stable, isotropic clear colloidal dispersion of oil, water and surfactant, frequently in combination with a cosurfactant which have high stability, ultralow interfacial tension, large interface area, low viscosity, and ease of preparation (2, 3). Conventional microemulsions can be categorized to oil-in-water, (O/W), water-in-oil (W/O), and bicontinuous phase microemulsions. Some advantages provided by microemulsions include an increase in solubility, especially for poorly soluble drugs and bioavailability protection of the unstable drugs against environmental conditions, and shelf life. Dexamethasone is a glucocorticoid drug prepared from artificial origin (4) is broadly used in inflammatory state and improvement of the normal immune response (5), congenital adrenal hyperplasia, nausea, and vomiting, particularly related with a rich dose of anticancer agents, respiratory disorders, skin disorders, (4) and rheumatism. The goal of this work was to develop and evaluate a microemulsion system for dexamethasone (0.1%, W/W) for topical delivery.

## 2. Objectives

The purpose of the present research was to formulate a transparent microemulsion as a topical delivery system for dexamethasone by mixing appropriate amount of surfactant including Tween 80 and Labrasol, cosurfactant such as capryol 90, and oil phase including labrafac lipophile wl-transcutol P (10:1 ratio) which was obtained from pseudoternary phase diagram.

## 3. Materials and Methods

Dexamethasone powder was purchased from Darou pakhsh company (IR Iran). Tween 80 and PG were obtained from Merck (Germany), Caprylocaproyl macrogoglycerides (Labrasol), Diethylene glycol monoethyl ether (Transcutol P), labrafac lipophil Wl 1349 and capryol 90 were gifted from GATTEFOSSE Company (France). All chemicals and solvents were of analytical grade. Fresh double distilled water was used in the experiments. Minitab15 software was used for experimental design and the evaluation of the effect of variables on responses. Sigma plot 11 software was used for providing tertiary phase diagrams.

### 3.1. Dexamethasone Assay

The quantitative determination of dexamethasone was performed by UV spectrophotometry (BioWave II, WPA) at λ_max_ = 244 nm.

### 3.2. Solubility of Dexamethasone

Solubility of dexamethasone was determined in different oil (labrafac lipophil wl 1349, transcutol-P, olive oil, almond oil, labrafac lipophil PG), surfactants (Labrasol, Tween 80) and cosurfactant (Capryol 90, Isopropyl alcohol) by dissolving an excess amount of dexamethasone in 3 mL of oil, surfactant and cosurfactant using a stirrer at 37˚C ± 0.5 for 72 h (6). The equilibrated samples were then centrifuged at 10000 rpm for 30 min to remove the undissolved drug. The solubility of dexamethasone was calculated by analyzing the filtrate spectrophotometrically using nanospectrophotometry (Biochrom WPA Bioware) after dilution with methanol at 244 nm.

### 3.3. Pseudoternary Phase Diagram Construction

To obtain concentration range of components for the existing boundary of microemulsion, pseudoternary phase diagrams were constructed using the water titration method (7). Three phase diagrams were prepared with the 1:1, 2:1, and 4:1 weight ratios of (Labrasol/Tween 80) Capryol 90 respectively. Oil phase (labrafac lipophil wl-Transcutol-P) and the surfactant mixture were then mixed at the weight ratios of 1:9, 2:8, 3:7, 4:6, 5:5, 6:4, 7:3, 8:2, and 9:1 (8). These mixtures were diluted dropwise with double distilled water, under moderate agitation. The samples were classified as microemulsions when they appeared as clear liquids (9). Several parameters influence on final properties of microemulsions. Full factorial design was used concerning with 3 variables at 2 levels for formulations. Major variables take part in determination of microemulsion’s properties include surfactant/cosurfactant ratio (S/C), percentage of oil (%Oil), and water percentage (%W). Eight different formulations with low and high values of oil (12% and 30%), water (10%, 15%), and S/Co mixing ratio (1:1, 4:1) were prepared for preparing microemulsion formulation.

### 3.4. Preparation of Dexamethasone Microemulsions

Various MEs were selected from the pseudoternary phase diagram with 1:1, and 4:1 weight ratio of Labrasol/Tween 80/Capryol 90 (Table 1). Dexamethasone (0.1%) was added to oil phase, then adding S/CoS mixture and an appropriate amount of double distilled water was added to the mixture drop by drop and the MEs containing dexamethasone were obtained by stirring the mixtures at ambient temperature (10, 11). 

**Table 1.** Composition of Selected Microemulsions

Formulation	% S + C	Oil, %	Water, %	(S:C)
**MED-1**	55	30	15	4:1
**MED-2**	60	30	10	4:1
**MED-3**	73	12	15	4:1
**MED-4**	78	12	10	4:1
**MED-5**	55	30	15	1:1
**MED-6**	60	30	10	1:1
**MED-7**	73	12	15	1:1
**MED-8**	78	12	10	1:1

### 3.5. Differential Scanning Calorimetry 

Differential scanning calorimetry (DSC) measurements were performed by means of a Metller Toldo DSC1 star ® system equipped with refrigerated cooling system (Hubert Tc45). Approximately 5-10 mg of microemulsion samples were weighted into hermetic aluminum pans and quickly sealed to prevent water evaporation from microemulsion samples. Simultaneously an empty hermetically sealed pan was used as a reference. Microemulsion samples were exposed in a temperature ranging from + 30˚C–50˚C (scan rate: 10˚C/min). To ensure accuracy and repeatability of data, DSC instrument was calibrated and checked under the conditions of use by indium standard. Changes of enthalpy quantities (∆H) were calculated from endothermic and exothermic transitions of thermograms by Equation 1 (11, 12):

**Equation 1.** ∆H = Peak area/Sample weight

### 3.6. Scanning Electron Microscopy 

Scanning electron microscopy (SEM) was used to characterize microstructure of micro emulsions. SEM of samples were analyzed by LEO 1455VP, Germany.

### 3.7. Zeta Potential Determination

Zeta potential of samples were measured by Zetasizer (Malvern instrument 1td ZEN3600, UK). Samples were placed in clear disposable zeta cells, and results were recorded. Before putting the fresh sample, cuvettes were washed with methanol and rinsed using the sample to be measured before each experiment (13).

### 3.8. Particle Size Measurements

The average droplet size of samples was measured at 25˚C by SCATTER SCOPE 1 QUIDIX (South Korea) and their refractory indices were also calculated (13).

### 3.9. Viscosity Measurements

The viscosity of microemulsions was measured at 25˚C with a Brookfield viscometer (DV-II + Pro Brookfield, USA) using spindle no. 34. With shear rate 100 rpm (14).

### 3.10. Conductivity Measurements

The electric conductivity of ME was measured with a conductivity meter (Metrohm Model 712). This was performed by using conductivity cell (with a cell constant of 1.0) consisting of two platinum plates separated by desired distance and having liquid between the platinum plates acting as a conductor (15).

### 3.11. pH Determination

The pH value of microemulsions was determined at 25˚C by pH meter (Mettler Toledo seven easy, Switzerland). All measurements were performed in triplicate.

### 3.12. Stability Study

The stability of microemulsions was studied regarding the temperature stability and centrifugation. Microemulsions were kept in various temperature conditions (4˚C, 25˚C and 37˚C), and observed for phase separation, flocculation or precipitation. Also, Microemulsions were centrifuged by High Speed Brushless Centerifuge (MPV-350R, POLAND at 10000 rpm for 30 minutes at 25˚C, and inspected for any change in their homogeneity.

### 3.13. Release Study

Franz diffusion cells (area 3.4618 cm^2^) with a cellulose membrane were used to determine the release rate of dexamethasone from different microemulsion formulations. The cellulose (molecular weight G12 000) membrane was first hydrated in double distilled water solution at 25˚C for 24 hours. The membrane was then clamped between the donor and receptor compartments of the cells. Diffusion cell was filled with 25 mL of phosphate buffer (pH = 7.4) and methanol (1:2). The receptor fluid was constantly stirred by externally driven magnetic bars at 300 rpm throughout the experiment. Dexamethasone microemulsion (5 g) was accurately weighted and placed in donor compartment. At 0.5, 1, 2, 3, 4, 5, 6, 7, 8, and 24 h time intervals, 2 mL sample was removed from receptor for spectrophotometric determination and replaced immediately with an equal volume of fresh receptor solution. Samples were analyzed by UV visible spectrophotometer (BioWaveII, WPA) at 244 nm. The results were plotted as cumulative released drug percent versus time. Drug release from MEs has been described by fitting on kinetic models in which commonly used such as zero order, first order, second order, 3/2 root of mass, linear and log wagner, Hixson-crowell, weibull, higuchi, korsmeyer - peppas models, and the model with higher r^2^ was selected.

### 3.14. X-Ray Diffraction 

X-Ray diffraction of dexamethasone microemulsion was performed using a Philips PC-APD diffractometer (Xpert MPD) with Goniometer type pW3050/e-2e. Ni-filtered Co Kα radiation (d = 1.78897˚A) at operating power® generator 40KeV and 30 mA was used. 2θ scans were made for all microemulsion formulations. The rang of XRD measurement was usually from 1.11 to 9.9˚2θ with scanning rate of 0.02˚/sec. The microemulsion samples were transferred to a spinner stage in a thermally controlled sample holder centered in the X-radiation beam. The scattering intensities data were collected at miniprop detector. All x-ray scattering were performed at 25˚C (16).

### 3.15. Statistical Methods

All the experiments were repeated three times, and data were expressed as the mean value ± SD. Statistical data were analyzed by one-way analysis of variance (ANOVA), and P < 0.05 was considered to be significant with 95% confidence intervals.

## 4. Results

### 4.1. Solubility of Dexamethasone

The maximum solubility of dexamethasone was found in labrafac lipophile wl:Transcutol P (10:1) (9.21 ± 0.13 mg/mL) as compared to other oils (Table 2). In addition, the highest drug solubility of dexamethasone in surfactants was found in Labrasol (8.49 ± 0.13 mg/mL), and Tween 80 (6.27 ± 0.12 mg/mL) and cosurfactant, capryol 90 (9.93 ± 0.27 mg/mL). The solubility of dexamethasone was found in oils, surfactants, and cosurfactants (Table 2). 

**Table 2.** Solubility of Dexamethasone in Various Oils, Surfactants and Cosurfactants (Mean ± SD, n = 3)

Phase Type	Excipient	Solubility
**Oil**	Labrafac lipophil wl 1349/Transcutol-P	9.21 ± 0.13
	Labrafac lipophil wl 1349	7.32 ± 0.09
	Olive Oil	4.12 ± 0.16
	Almond Oil	5.25 ± 0.27
	Labrafac lipophil PG	9.05 ± 0.04
**Surfactant**	Tween 80	6.27 ± 0.12
	Labrasol	8.494 ± 0.13
**Cosurfactant**	Capryol 90	9.93 ± 0.27
	Isopropyl Alcohol	5.13 ± 0.15

### 4.2. Phase Studies

**Figure 1.**
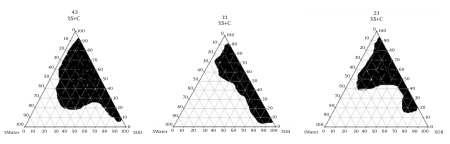
The Pseudoternary Phase Diagrams of the Oil-surfactant/Cosurfactant Mixture-water System at the 1:1, 2:1, and 4:1 Weight Ratio or Labrasol/Tween 80/Capriol 90 at Ambient Temperature, Dark Area Show Microemulsions Zone

The aim of construction of pseudoternary phase diagrams was to find out the existence region of microemulsions. The Pseudoternary phase diagrams of labrafac lipophil wl, Transcutol P (10:1)/Labrasol-Tween 80/capryol 90/Water are presented in Figure 1. 

### 4.3. Characterization of the Dexamethasone–Loaded Microemulsion Preparations

Eight different MEs were selected from the pseudoternary phase diagram with 1:1, and 4:1 weight ratio of Labrasol-Tween 80/Capryol90. The composition of microemulsions is shown in Table 1. The viscosity, pH, mean particle size, conductivity, zeta potential, refractive index, and polydispersity index (PI) of Dexamethasone microemulsions are illustrated in Table 3. The ME formulations in this study showed the average viscosity range (57 ± 0.74 cps–206 ± 1.24 cps), zeta potential (-0.177 to -3.41 mv), pH value (7.157 ± 0.07), and particle size (5.09 ± 1.2-159 ± 7.2 nm). 

**Table 3.** pH, Viscosity, Particle Size, Conductivity, Zeta Potential, Refractive Index and PI of Selected Dexamethasone Microemulsions (Mean ± SD, n = 3)

Formulation	pH	Viscosity, cps	Mean Particle Size, nm	Conductivity, ms/cm	Zeta Potential, mV	Refractive Index	Polydispersity Index
**MED-1**	7.10 ± 0.72	69 ± 1.08	11.3 ± 2.1	0.0881 ± 0.0021	-0.808	1.441 ± 0.41	0.363 ± 0.25
**MED-2**	7.04 ± 0.41	91 ± 1.05	16.3 ± 3.5	0.0810 ± 0.0015	-1.35	1.443 ± 0.53	0.358 ± 0.22
**MED-3**	7.15 ± 1.32	184 ± 0.61	14 ± 2.8	0.0864 ± 0.0025	-3.24	1.441 ± 1.64	0.363 ± 0.1
**MED-4**	7.22 ± 0.58	206 ± 1.24	159 ± 7.2	0.0668 ± 0.0017	-3.41	1.446 ± 1.12	0.366 ± 0.2
**MED-5**	6.41 ± 0.77	57 ± 0.74	7.16 ± 2.3	0.0852 ± 0.0022	-2.41	1.443 ± 1.86	0.363 ± 0.1
**MED-6**	6.56 ± 1.12	70 ± 0.44	9.37 ± 3.1	0.0693 ± 0.0004	-0.331	1.445 ± 0.82	0.353 ± 0.1
**MED-7**	6.73 ± 0.92	177 ± 0.57	6.41 ± 2	0.0706 ± 0.0002	-0.177	1.444 ± 0.49	0.363 ± 0.2
**MED-8**	7.10 ± 0.67	159 ± 0.83	5.09 ± 1.2	0.0893 ± 0.0014	-1.63	1.447 ± 1.25	0.363 ± 0.18

**Figure 2.**
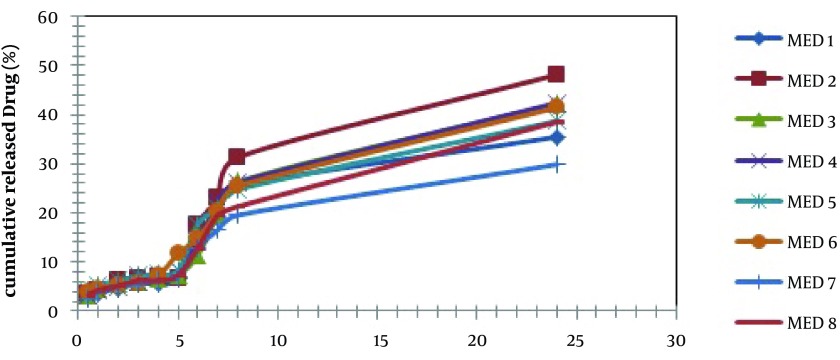
In vitro Release Profile of MEs Formulation of Dexamethasone

**Figure 3.**
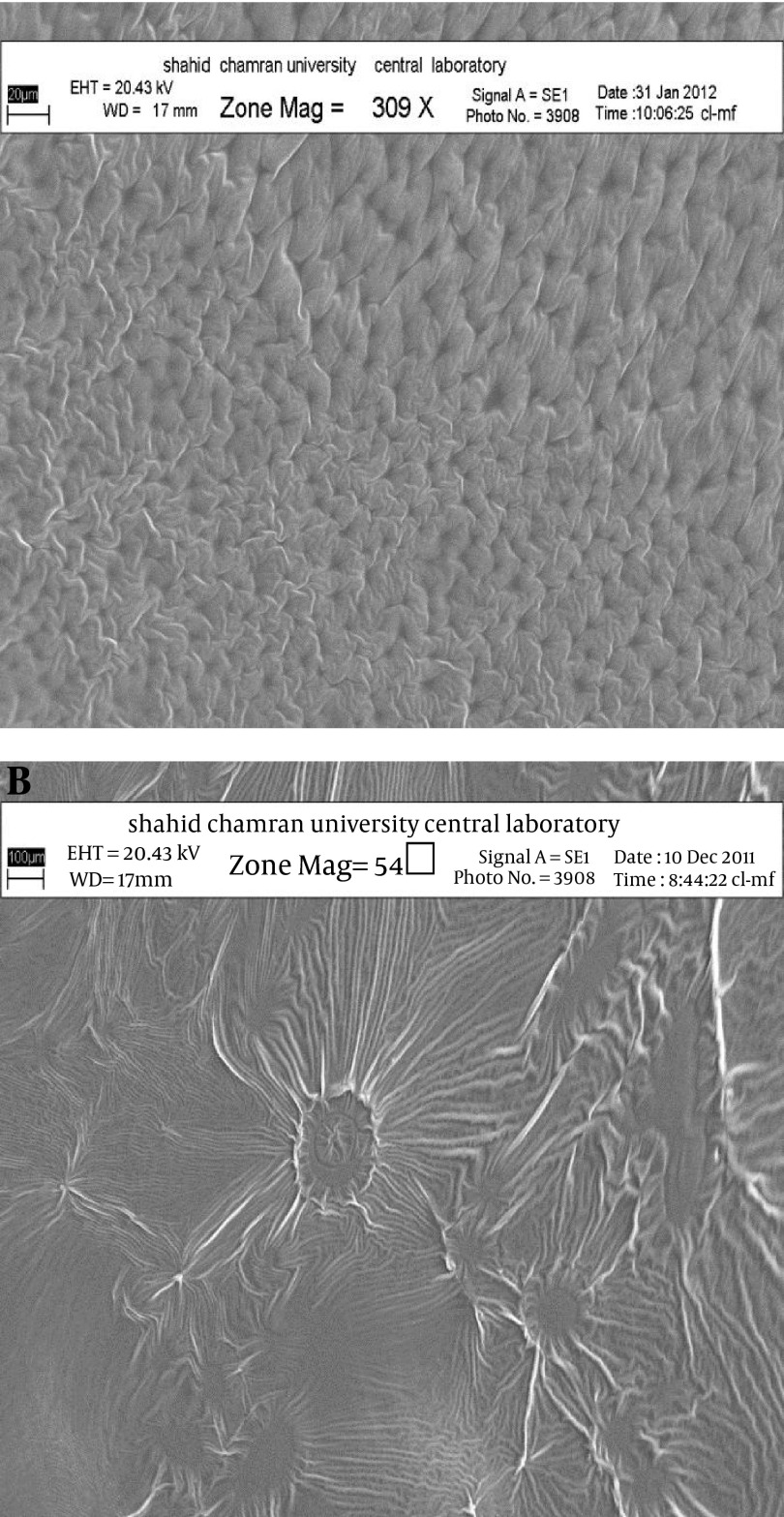
A. SEM Photographs of ME-3 and B. ME-7

**Figure 4.**
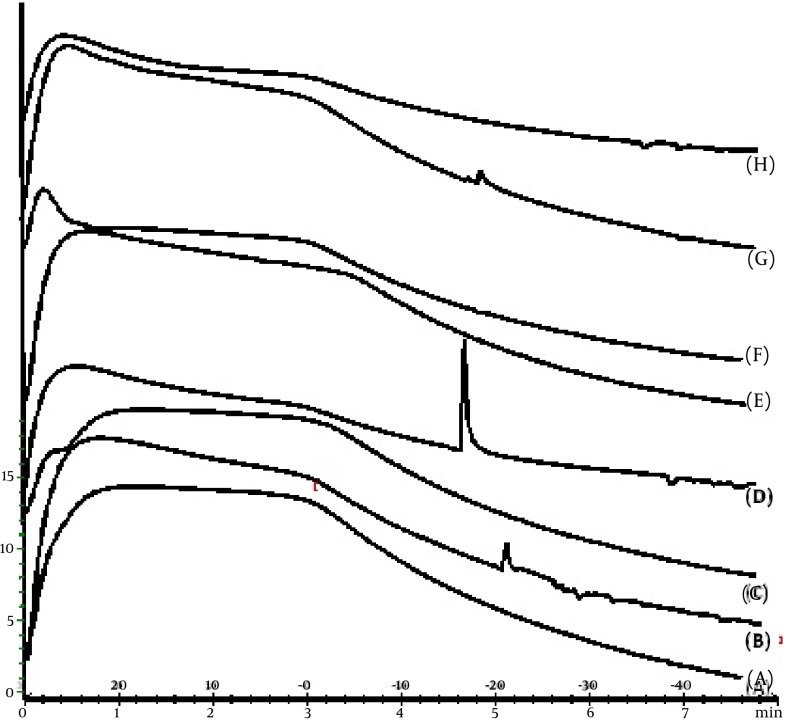
A. DSC Cooling Thermograms of MED-1, B. MED-2, C. MED-3, D. MED-4, E. MED-5, F. MED-6, G. MED-7, H. MED-8

Figure 2 shows the release profiles of dexamethasone microemulsions. Drug release profile showed that 48.18% of the drug released in the 24 hours of experiment for MED-2. In all formulations, release profile is two parts. It seems that higher drug solubility in oil phase of water in oil MEs has caused burst release in the first times, while constant release in the last times is belonged to slow drug release from internal phase of MEs. Drug released percent and kinetic of release in selected microemulsions are displayed in Table 4. There was second order kinetic for MED-2. Figure 3 shows the SEM images of MED-2 and MED-7. Figure 4 represents DSC cooling thermograms of dexamethasone MEs. In cooling curves of the samples, bulk water (free water) and bound water are obtained in 0 to -5.7˚C and -15 to -41˚C, respectively. 

**Table 4.** Percent Release and Kinetic Model Release of Selected Microemulsions (Mean ± SD, n=3)

Formulation	Release, %	Kinetic Model Release	R^2^
**MED-1**	35.49	Pepas	0.9736
**MED-2**	48.18	Second order	0.9773
**MED-3**	42.23	3/2 root of mass	0.9804
**MED-4**	42.41	Second order	0.9810
**MED-5**	38.73	Pepas	0.9824
**MED-6**	41.47	Second order	0.9902
**MED-7**	29.87	Pepas	0.9798
**MED-8**	38.54	Pepas	0.9785

With X-ray scattering experiments, characteristic interferences are generated from an ordered microstructure.

**Figure 5.**
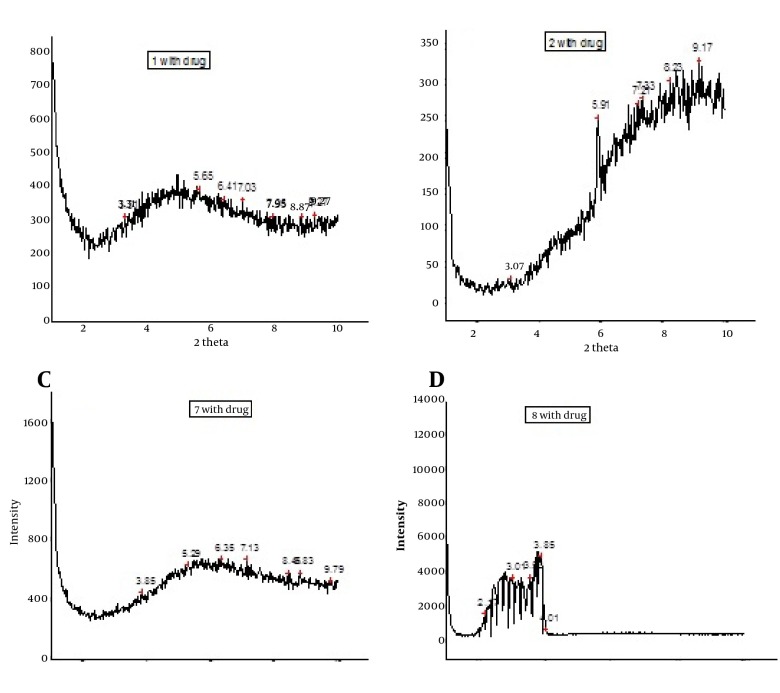
A & B. XRD Peaks of MED-1 and MED-2, and C & D. MED-7 and MED-8

The XRD peaks of MED-1, MED-2, MED-7, and MED-8 formulations are displayed in Figure 5. These results indicate lamellar, cubic, and hexagonal microstructures in various formulations. The visual inspection test was performed for 3 months by drawing ME sample at weekly interval for the first month, and monthly interval for the subsequent months. The visual observation showed no evidence of phase separation or any flocculation or precipitation. These samples also showed no sign of phase separation under stress when subjected to centrifugation at 10000 rpm for 30 min. The centrifugation tests revealed that microemulsions were remained homogenous without any phase separation throughout the test, which indicates good physical stability of both preparations. 

## 5. Discussion

To develop microemulsion formulations the optimum oil was selected by determining the concentration of dexamethasone that would dissolve. Based on the solubility studies of dexamethasone in oil, surfactant and cosurfactant and the preformulation studies we found that labrafac lipophil wl-Transcutol P, labrasol, Tween 80 and capryol 90 could be the most appropriate combinations for preparation of microemulsion. It seems that phase behavior depended on surfactant and cosurfactant properties. The weight ratio of surfactant/cosurfactant is a critical and important parameter affecting phase behaviors of microemulsion. The extent of microemulsion area increasing with increasing relative concentration of surfactant was reported (11). The phase diagrams revealed that microemulsion region extended with large amount in the weight ratio of surfactant/cosurfactant (km = 2-4). Phase diagrams indicated more width microemulsion region with a rise in S/C ratio. The ME formulations had the mean particle size in the range of 5.09-159 nm. The MED-8 formulation had the lowest average particle size 5.09 ± 0.14 nm with polydispersity index (PI) 0.365 ± 0.18. It seems that the mean particle size is decreased with less percentage of oil phase in some of MEs. The refractive index (RI) of the ME formulations was found 1.44 which is near to oil phase indicating that MEs formulations have water -in-oil structures. Analysis of variance showed that correlation between RI and independent variables (%W) is not significant (P > 0.05). Electrical conductometry is a helpful tool to evaluate conductive behavior of microemulsion samples. Due to the conductivity properties of aqueous phase, O/W microemulsions demonstrate higher conductivity values than the W/O microemulsions (15). The conductivity of Dexamethasone samples was in the range of 0.0693-0.0893 ms/cm. The ME formulations had appropriate observed pH value (7.157 ± 0.07) which is best for topical application. Incorporation of dexamethasone did not significantly affect the observed pH value of the ME formulations. The ME formulations had the average viscosity range (57 ± 0.74 cps - 206 ± 1.24 cps). The highest viscosity belongs to MED-4 formulation with bicontinuous structure. Multivariate regression was applied for the analysis of correlation between independent variables and MEs viscosity. The percent of water, S/C and oil percent had more positive and negative effects on viscosity, respectively. The ME formulations had the zeta potential average (-0.177 to -3.41 mv). The highest zeta potential belongs to MED-4 formulation with micellar and bicontinuous structure, and the lowest belongs to MED-7 with bicontinuous structure. Multivariate regression was used for the analysis of correlation between independent variables and MEs zeta potential. The S/C had more positive effect on zeta potential. There was no significant difference between the zeta potential and independent variables (P > 0.05). The cumulative amount of dexamethasone that had permeated through the cellulose membrane (%) was plotted as a function of time (hours). In this study, MED-2 and MED-7 had the highest and lowest accumulative release percent, respectively. There was no correlation between the water percent, S/C ratio and release percentage values of dexamethasone (P > 0.05). Multivariate regression was used for the analysis of correlation between independent variables and MEs release. The percent of oil and S/C had more positive effect, and the water percent had negative effect on release percent of dexamethasone MEs. This study demonstrated that physicochemical properties and in vitro release were dependent upon the contents of S/C, water and, oil percentage in formulations. It seems that the effect of capryol 90 as cosurfactant in MEs release profile may be as retardant, since with decrease in S + C and water, and increase in oil phase, percent could be obtained high in vitro percentage release. Table 4 represents release percent and kinetic of release of selected ME formulations. DSC results indicate important information about water state in microemulsions (16). The water mixed in the microemulsion systems can be either bound (interfacial) or bulk (free) state. In cooling curves of the sample MED-1, DSC thermograms showed one exothermic peak at around -10C which indicates that the freezing of free water in this formulation and inMED-2 implies two exothermic peaks at around -1˚C (bulk water) and -20˚C (bound water). In cooling curves of MED-3, DSC thermograms showed two exothermic peaks at -1.7˚C and -19˚C (bound water), and one exothermic peak at -40.7˚C for ME D-4 which indicates bound water. Since the freezing temperature is very low, water must be strongly bound or interacted with surfactants (7). DSC thermograms of MED-5 and MED-6 showed two exothermic peaks at -5.7˚C (bulk water) and -2˚C (bulk water) respectively. In cooling curves of MED-7, DSC thermograms represented two exothermic peaks at -0.4˚C, -22.4˚C, which indicates free water and oil phase freezing, respectively. DSC thermograms of MED-8 showed two peaks at 0˚C (bulk water) and -36.9˚C (bound water) (17). Figure 4 shows DSC cooling thermogram of MED formulations. Pure oil peaks could be observed at -20 and -28˚C, and became smaller when the water concentration increased. The peaks disappeared at 15% wt/wt water, suggesting that oil phase changed from external to internal phase (7). Small-angle X-ray Scattering (SAXS) techniques have been used by several researchers to obtain information about droplet size and microstructure of microemulsions (7, 17, 18). With X-ray scattering experiments, characteristic interferences are generated from an ordered microstructure. A typical interference pattern arises due to specific repeat distances of the associated interlayer spacing d. by Bragg’s equation. The periodic interlayer spacing (d) was calculated by the Bragg’s equation n λ = 2dsinθ. SAXD is important for the exact determination of the distances of d of liquid crystalline. The SAXD method does not only calculate interferences between the periodic interlayer spacings, but also can determine the sequence of the interferences the type of liquid crystal (19, 20). The effect of dexamethasone and independent variables on scattering property and internal structuration of the microemulsions was investigated. For MED formulations, bicontinuous phase was recognized for formulations 3.4.7, 8 which include approximately equal volume of oil and water. Bicontinuous structure is proved by SEM micrograph. With decreasing surfactant/cosurfactant amount in MED-7, MED-8, lamellar structures (MED-3, MED-4) changed to cubic structures. This effect of surfactant concentration on microstructure of microemulsions was reported previously by Strey (21). Reverse Hexagonal structure was established in MED-2 and MED-6 with the highest oil/surfactant ratio. It seems that high amount of oily phase/surfactant ratio produced high-ordered structure. This finding is in contrast with previous study that indicated destabilising effect of oil on hexagonal structure (22). The effect of s/c and dexamethasone on microstructures was found significant. On the other hand, water amount, surfactant concentration, and oil/surfactant ratio displayed significant effects on microstructures. Dexamethasone converts microstructures from cubic to hexagonal in MED-1, MED-2 MED-8. SAXS technique and SEM obtained important information about microstructure of microemulsions. W/O and bicontinuous microemulsions with different microstructures were found in formulations. Internal structure of microemulsion is impressed by surfactant concentration, oil/surfactant ratio, amount of water, and physicochemical properties of cosurfactants. In conclusion, microemulsions make good solubility of dexamethasone with vast range of microstructures. ME-2 may be preferable for topical dexamethasone formulation; however, more studies are still needed to be performed to elucidate the mechanisms of drug delivery into the skin.
